# Exciton Relaxation Dynamics in Photo-Excited CsPbI_3_ Perovskite Nanocrystals

**DOI:** 10.1038/srep29442

**Published:** 2016-07-12

**Authors:** Qinghui Liu, Yinghui Wang, Ning Sui, Yanting Wang, Xiaochun Chi, Qianqian Wang, Ying Chen, Wenyu Ji, Lu Zou, Hanzhuang Zhang

**Affiliations:** 1 Femtosecond Laser Laboratory, Key Laboratory of Physics and Technology for Advanced Batteries (Ministry of Education), College of Physics, Jilin University, Changchun 130012, P. R. China

## Abstract

The exciton relaxation process of CsPbI_3_ perovskite nanocrystals (NCs) has been investigated by using transient absorption (TA) spectroscopy. The hot exciton relaxation process is confirmed to exist in the CsPbI_3_ NCs, through comparing the TA data of CsPbI_3_ NCs in low and high energy excitonic states. In addition, the Auger recombination and intrinsic decay paths also participate in the relaxation process of CsPbI_3_ NCs, even the number of exciton per NC is estimated to be less than 1. Excitation intensity-dependent TA data further confirms the existence of Auger recombination. Meanwhile, the spectral data also confirms that the weight of hot exciton also increase together with that of Auger recombination at high excitation intensity when CsPbI_3_ NCs in high energy excitonic states.

The materials with nanoscale size exhibit many interesting characteristics, especially in the optoelectronic fields. Many optoelectronic devices based on nanocrystals have been prepared, such as light-harvesting and -emitting devices. With the development of synthesis techniques, many materials have been used to prepare nanocrystals, which opened a unique window for people to understand the world. Meanwhile the performance of optoelectronic devices based on nanocrystals have also improved continuously. Recently, a new family of nanocrystals has attracted tremendous world-wide attention. These all inorganic cesium lead halide perovskites (CsPbX_3_, X = Cl, Br, I or their mixed halide systems) nanocrystals exhibit excellent optical properties such as narrow emission line-width, tunable emission over entire visible spectrum, large optical absorption cross section, high quantum yields, and long carrier diffusion length and mobility. Apparently, the lead-halide perovskite nanocrystals exhibit a huge potential application in the light-emitting diodes^1^, electroluminescence devices^2^, low-threshold lasers^3^,^4^ and sing-photon source^5^. Up to now, researchers have started focusing on the ultrafast dynamic processes occurring in perovskite materials, such as the charge carrier diffusion^6^, hot carriers relaxation, Auger recombination^7^,^8^ process and interfacial charge transfer^9^,^10^ which provides clear evidence that the free-carrier model is more suitable to interpret the optical properties of bulk perovskite materials^11^ in comparison with the exciton model. The dynamic processes in perovskite materials are also sensitive to the structure^12^, chemical components^13^, detection positions^14^,^15^, and their surrounding environment^16^. If the perovskite materials are confined in a nano-scale space, such as two-dimensional quantum wells^17^ and perovskite nanocrystals^18^,^19^, their relaxation processes become more dependent on the carrier recombination mechanism due to the limited scale. Although the understanding of ultrafast excitons and carriers dynamic processes have obtained noticeable improvement, the understanding about the nature of photo-excitation across the bandgap in perovskite nanocrystals remains limited. Moreover, dynamic behaviors of corresponding transient species require to be further explored. In this paper, we investigate the photoexcitation dynamic processes of CsPbI_3_ perovskite NCs in low and high energy excitonic states by using transient absorption spectroscopy with different pump photon energy. This provides a clear picture of the exciton relaxation processes occurring in inorganic perovskite NC.

## Experiment

Steady-state absorption measurements were carried out on a UV–Vis spectrophotometer (Purkinje, TU-1810PC). Fluorescence spectra were recorded by a fiber optic spectrometer (Ocean Optics, USB4000) with excitation pulse at 400 nm. The fluorescence dynamics are collected by Time-correlated single photon counting (TCSPC)^20^ setup. The femtosecond transient absorption (TA) technique is introduced in Support Information. The excitation pulse is generated from optical parameters amplifiers (Coherent, TOPAS), whose photon energy is set up to 2.53 and 1.90 eV. Typical transmission electron microscopy (TEM) images were taken on a FEI Tecnai G2 F20 with an accelerating voltage of 200 kV. All the measurements were performed at room temperature.

## Results and Discussion

**Figure 1:**
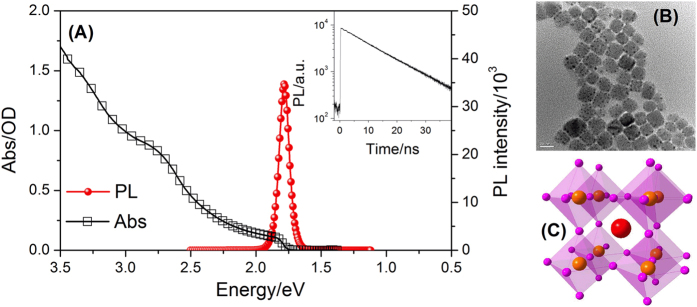
(**A**) Steady absorption and fluorescence spectra of CsPbI_3_ NCs; (**B**) Typical TEM images of CsPbI_3_ NCs; (**C**) the structure of CsPbI_3_ perovskite NCs (Pink spheres: I; Red spheres: Cs; Orange spheres: Pb). Inset: the fluorescence trace of CsPbI_3_ NCs detected by TCSPC.

As depicted in Fig. 1(A), the ground state absorption spectrum of CsPbI_3_ perovskite NCs shows two small absorption peaks, which are located at 660 nm near the long-wavelength edge, and at 480 nm, which is on the interband absorption background. They may correspond to low energy and high energy excitonic absorption band. The similar absorption bands were observed in the absorption spectra of CsPbI_3_ film in the previous report^21^,^22^. The fluorescence peak is located at ~700 nm and the shape of the fluorescence spectrum is independent of the time elapsed after excitation, which is similar to that of CH_3_NH_3_PbI_3_ film^6^. Its dynamic trace at fluorescence peak is presented in the inset of Fig. 1(A), whose estimated lifetime is ~12.3 ns. According to our TEM results shown in Fig. 1(B), as-obtained perovskite NCs display a relative mean size distribution (~18 ± 2 nm) with cubic shape, which reflects its structural nature of cubic lattice in Fig. 1(C). This size is much larger comparable to the excitonic Bohr radius (~7 nm)^23^. However, the photo-generated exciton are also limited in the NCs and could not separate free carriers. The fluorescence of CsPbI_3_ perovskite NCs should be assigned to the exciton recombination, and is different from that of bulk materials with large scale, which is attributed to the recombination of electrons and holes^24^.

**Figure 2:**
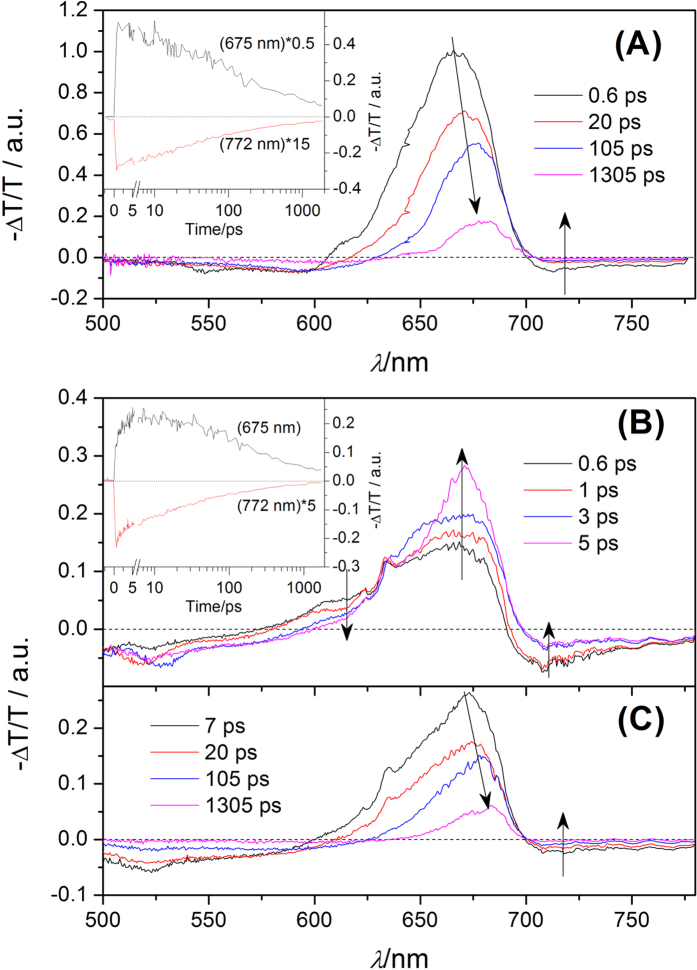
Time-dependent transient spectra of CsPbI_3_ NCs at low (**A**) and high (**B**) energy excitonic states. Inset: The corresponding wavelength dependent traces of CsPbI_3_ NCs.

**Figure 3:**
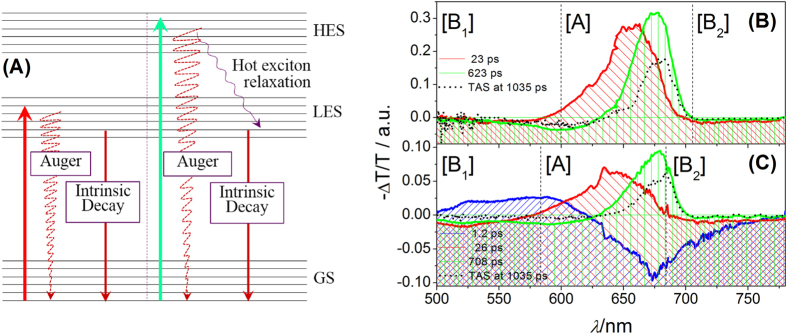
(**A**) The exciton relaxation mechanism of CsPbI_3_ perovskite NCs; (**B**) the spectral component of CsPbI_3_ perovskite NCs from global fitting at low (**B**) and high (**C**) energy excitonic states. The red, green, blue spectra correspond to the Auger recombination, intrinsic decay and hot exciton. All of them are divided into three parts: [B_1_], [A] and [B_2_], which are used to further confirm their properties in the following sections.

As seen in Fig. 2(A), we present ultrafast dynamics in CsPbI_3_ NCs at low energy excitonic state (LES), which is excited by pump pulses with energy of 1.90 eV. Meanwhile, the excitation intensity is adjusted so as to ensure the number of exciton per nanocrystal is estimated to be ~0.63. According to the steady absorption spectra, the TA spectra of CsPbI_3_ NCs on LES shows three main spectral features: two broad negative bands spanning in the visible region, located around 575 and 725 nm, and a broad positive band in the range of 600–700 nm. The broad positive band are attributed to the superposition of ground state photo-bleaching (GSPB) and simulated emission (SE) due to the close resemblance to the spectra of steady absorption and photoluminescence; the negative spectral feature is assigned to photo-induced absorption of excited state (EA). Note that the broad positive band shows a redshift with time, indicating that there are multi-relaxation pathways in the relaxation process from the LES. Inset of Fig. 2(A) shows the dynamic traces of broad band at 670 nm and that of EA at 780 nm. Both of them show a similar monotonic decay behavior, but their relaxation rate is different. As a result, the TA spectrum of CsPbI_3_ NCs on LES can be nicely fitted using a model with two components having decay times of 23 ps, and 623 ps based on global analysis, and the corresponding spectra are seen in Fig. 3(B). Considering the number of exciton per nanocrystal is not low enough and the Auger recombination process could still participate in the relaxation process of LES, we believe that the spectral components with lifetime of 23 ps should be attributed to the contribution of Auger recombination. Meanwhile, it is also found that the TA spectra at ~1.3 ns is very similar to the spectral component with long lifetime. In addition, the scale of nanocrystal is very large, the quantum confinement effect is very weak as mentioned. The recombination originated surface state could not play any role in the relaxation process. Therefore, it is suitable to consider that the spectral components with lifetime of 623 ps is assigned to the intrinsic decay with long lifetime. Finally, the transient absorption data exhibits that the CsPbI_3_ NCs on LES is able to relax on the ground state through Auger recombination process and the intrinsic decay paths after photo-excitation. The relaxation mechanism of CsPbI_3_ NCs pump by the pulse with photon energy of 1.90 eV are summarized in Fig. 3(A).

The exciton dynamic process of CsPbI_3_ NCs on high energy excitonic state (HES) excited by pump pulse with photon energy of 2.53 eV are presented on Fig. 2(B). Herein, the number of exciton per nanocrystal is also adjusted to be about 0.7, through changing the excitation intensity. Apparently, the structure of TA spectra of CsPbI_3_ NCs in HES is similar to those in LES, but the corresponding time evolution is clearly different. In Fig. 2(B), the amplitude of broad positive band located at about 675 nm gradually increases from 0.6 to 5 ps, meanwhile the short wavelength part of positive band shows a tendency to decrease. In such a situation, it is suitable expected that the rising signal at ~670 nm should be assigned to the evolution of SE and the decreasing signal at 615 nm should be attributed to the contribution of GSPB. During this time, the position of positive band is also invariable. However, the positive signal gradually becomes narrower, since the GSPB and SE show different dynamic behaviors. This indicates that the CsPbI_3_ NCs exhibits an energy state dependent relaxation process. As given in Fig. 2(C), the amplitude of the whole spectra begins to weaken after 7 ps, and the corresponding positive signal also exhibits a redshift, which is similar to that occurring in Fig. 2(A). Considering the CsPbI_3_ NCs are on the HES, it is expected that the hot exciton relaxation could be responsible for the initial dynamic phenomenon. During the cooling of hot exciton, it is expected that the excess energy transfers to the phonon path^2^. During the cooling process of hot exciton, the population on LES increases due to the transfer from the HES, and leads to the rising behavior of SE signal in the broad positive band of TA spectra. Meanwhile, the evolution of positive signal near ~615 nm as shown in Fig. 2(B) exhibits that some transient species directly go back to the ground state. As mentioned above, we also expect that the Auger recombination could be responsible for the evolution of GSPB in the positive band, which is located near 615 nm. The time-dependent redshift of EA signal is reported in bulk CH_3_NH_3_PbI_3–x_Cl_x_ perovskite materials^2^, but this phenomenon is not observed in CsPbI_3_ NCs. Considering that the electron and hole in Mott-Wannier exciton is bound in NCs, they almost have no dipole moment and introduces no electric field^25^, which is different from that of free carrier (electron or hole) in bulk materials. After the CsPbI_3_ NCs decay on the LES through hot relaxation, it should follow the intrinsic decay path to go back to the ground state. Since the hot exciton cooling, Auger recombination and intrinsic decay path could participate in the relaxation process of CsPbI_3_ NCs in HES, the corresponding relaxation paths are summarized in Fig. 3(A). Herein, the TA spectra of CsPbI_3_ NCs in HES determined by global analysis exhibits a complicated relaxation dynamic process. As seen in Fig. 3(C), three components are required to obtain the best fit from the global analysis, and their lifetimes are 1.2, 26, 708 ps. The fastest components should correspond to the hot exciton relaxation of NCs from HES to LES^26^. The components with lifetimes of 26 and 708 ps are both similar to those excited by 650 nm laser in the spectral shape. It is suitable to speculate that they are also due to the contribution of the Auger recombination and the intrinsic decay. The TA spectra at 1.3 ns is also given in Fig. 3(C), and the similarity further confirm our speculation. Note that the Auger recombination process have happened during the process of hot exciton cooling which should compete with the hot exciton cooling after photoexcitation. The size of NCs in our experiment is so large that its properties are similar to those of bulk materials Therefore, the hot exciton cooling in CsPbI_3_ NCs should be ascribed to the hot-phonon bottleneck effect^27^.

**Figure 4:**
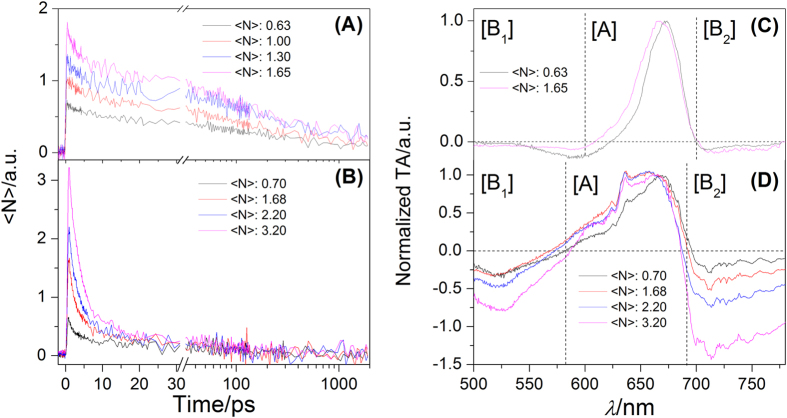
The TA curves at 780 nm of CsPbI_3_ NCs at low (**A**) and high (**B**) energy excitonic states, when the number of exciton per one NC increases; the <N> dependent normalized initial TA spectra of CsPbI_3_ NCs which is excited by 650 nm at 1.8 ps (**C**) and 490 nm lasers at 1.4 ps (**D**), respectively. In order to further analyze the spectral components in TA spectra, they are also divided into three parts: [B_1_], [A] and [B_2_], the positions of split points are all the same as those in Fig. 3(B,C).

The ES curves at 780 nm of CsPbI_3_ NCs distributed on HES and LES at different excitation intensities are presented in Fig. 4. As the excitation intensity increases, the average number of exciton per NCs could increases. The relaxation process of NCs on LES is dependent of the number of exciton per one NCs (<N>). As seen in Fig. 4 (A), the weight of fast relaxation component in the initial part of decay curves gradually enhances as <N> increases, which suggests that the Auger recombination is responsible for this fast relaxation component^28^. The <N> dependent TA spectra at 1.0 ps are presented in Fig. 4(C), which is normalized by the positive band in TA. As <N> increases from 0.63 to 1.67, the [B_1_] and [B_2_] part in the TA spectra vary in the amplitudes and the position of [A] part in TA spectra shows a little blue shift. As seen in Fig. 3(B), it is obvious that the peak shift of [A] part as <N> increases in Fig. 4(C) is attributed to the enhancement of the weight of red spectral component in the whole TA spectra. This also confirms that the red spectral component in Fig. 3(B) should be assigned to the contribution of Auger recombination. In addition, the red and green spectral components in Fig. 3(C) should actually correspond to the Auger recombination and intrinsic decay, since their spectral component and lifetime are both similar to those in Fig. 3(B). Since multiple relaxation pathways participate in the exciton relaxation process of CsPbI_3_ NCs on HES, it becomes more complex as presented in Fig. 4(B). The intensity-dependent fast recombination process still exists in the decay curves, the decay traces of CsPbI_3_ NCs from 60 to 2000 ps is almost invariable with change in <N>. This indicates that the NCs would quickly decay from HES through hot exciton cooling and Auger recombination, and then follows a similar decay path to go back to the ground state. The initial TA spectra at 1.4 ps are normalized and shown in Fig. 4(C), which exhibits that the [B_1_] and [B_2_] part in TA spectra is very sensitive to the change in <N>. As exhibited in Fig. 3(C), it is believed that the weight enhancement of blue spectral components in the whole TA spectra should be responsible for the variance of [B2] part in Fig. 4(D). In other words, the weight of hot exciton could also increase, as the <N> increases. As given in Fig. 3(C), the Auger recombination and intrinsic decay exhibit negative signals, and the hot exciton shows positive one. They are overlapped. Therefore, the intensity of [B_1_] part in Fig. 4(D) increase, but the variable amplitude is not larger in comparison with that of [B_2_] part. Apparently, the variance of [A] part in Fig. 4(D) is also attributed to the overlapping of multiple spectral components in this region and the variance of their weight when <N> increases. The Auger recombination and the hot exciton both together participate in the exciton relaxation process of CsPbI_3_ NCs at high excitation intensity. Their weight both increases, which could be responsible for the obvious enhancement of EA signal in [B_1_] and [B_2_] region.

## Conclusion

We have studied the exciton relaxation dynamics of CsPbI_3_ perovskite nanocrystals in low and high energy excitonic states by means of time-resolved transient absorption spectroscopy. Firstly, we revealed that the NCs on the high energy excitonic state have to relax on the LES through the hot exciton cooling after photoexcitation. Secondly, the NCs have to go back to LES, due to hot exciton relaxation and Auger recombination. The excitation-intensity TA curves further confirms that the Auger recombination still exists in the relaxation process of CsPbI_3_ perovskite nanocrystals, even though the number of exciton in nanocrystals is estimated to be ~0.67. The intensity-dependent normalized transient absorption spectra confirm the existence of Auger recombination and the weight of hot exciton could increase together with that of Auger recombination when the excitation intensity enhances. These results are beneficial for further investigation on the photophysical characteristics of pure inorganic perovskite nanocrystals.

## Methods

### Transient absorption spectroscopy

The femtosecond transient absorption spectroscopy was performed as follows. The laser pulse is generated from laser system (Coherent). The output of 2.5 mJ pulse energy, 120 fs pulse width, at 800 nm wavelength with a repetition rate of 1 K Hz was split into two parts. One of them was used to generated a white light continuum to be used as the probe beam by focusing the beam into a 2 mm water cell. The other was either used to pump optical parameters amplifier (Coherent, TOPAS) to generate excitation pulse at 490 nm (2.53 eV) and 650 nm (1.90 eV). These pulses were sent to a delay line and modulated by a synchronized optical chopper (Terahertz Technologies Inc., C-995) with a frequency of 500 Hz and were used as the pump beam to excited the sample. The time-dependent transient absorption spectra were recorded with a highly sensitive spectrometer (Avantes AvaSpec-2048 × 16). The polarization direction of the excitation and the probe beams was horizontal. The thickness of quartz cell is about 1 mm. The excitation spot is ~2 mm in diameter.

### Transient absorption test

We prepared CsPbI_3_ perovskite nanocrystals solution with concentration of 1.25 × 10^−4 ^mmol mL^−1^ (S-I, OD: 0.677 at 490 nm) and 3.12 × 10^−4 ^mmol mL^−1^ (S-II, OD: ~0.415 at 650 nm). The mole concentration of CsPbI_3_ nanocrystals are determined by the mass of lead measured via the inductively coupled plasma (ICP) atomic emission spectrometric analysis (POEMS, TJA). The absorption cross-section is estimated to be ~2.2 × 10^−15^ cm^2^ (at 650 nm), and 9.0 × 10^−15^ cm^2^ (at 490 nm). In order to confirm the accuracy of cross section of linear absorption, we also estimated the cross-section at 400 nm, and it is 2.0 × 10^−14^ cm^2^, which is similar to that estimated by transient photoluminescence in a recent work (2.5 × 10^−14^ cm^2^) and also close to that in ref. 29. The S-I and S-II are excited by pump pulse with photon energy of 2.53 (490 nm) and 1.90 eV (650 nm). Therefore, the distribution of pump light in quartz cell with S-I could be similar to that with S-II. The laser power at 490 nm is 0.14 mW and that at 650 nm is 0.23 mW, in order to ensure that the number of exciton per nanocrystal is similar to each other in the initial wavelength-dependent transient absorption measurement. In this situation, the exciton relaxation process of CsPbI_3_ perovskite nanocrystals LES and HES are compared in detail. In addition, the power of laser is also adjusted in order to change the number of exciton per one nanocrystal (<N>) and discuss the <N> dependent exciton relaxation process.

## Additional Information

**How to cite this article**: Liu, Q. *et al*. Exciton Relaxation Dynamics in Photo-Excited CsPbI_3_ Perovskite Nanocrystals. *Sci. Rep.* **6**, 29442; doi: 10.1038/srep29442 (2016).
